# Antibacterial Hydrogels for Wound Dressing Applications: Current Status, Progress, Challenges, and Trends

**DOI:** 10.3390/gels10080495

**Published:** 2024-07-26

**Authors:** Jie Zhu, Hongju Cheng, Zixian Zhang, Kaikai Chen, Qinchen Zhang, Chen Zhang, Weihong Gao, Yuansheng Zheng

**Affiliations:** 1 School of Textiles and Fashion, Shanghai University of Engineering Science, Shanghai 201620, China; zj910205@126.com (J.Z.); 18051920601@163.com (H.C.); 13462991136@163.com (Z.Z.); sxxxzhangqinchen@163.com (Q.Z.); gaoweihong@sues.edu.cn (W.G.); 09170001@sues.edu.cn (Y.Z.); 2 State Key Laboratory of Separation Membranes and Membrane Processes, School of Materials Science and Engineering, Tiangong University, Tianjin 300387, China; 3 Shanghai Science and Technology Exchange Center, Shanghai 200030, China

**Keywords:** antibacterial hydrogel, wound dressing, infection treatment, antibacterial therapies

## Abstract

Bacterial infection treatment for chronic wounds has posed a major medical threat and challenge. Bacteria at the wounded sites can compete with the immune system and subsequently invade live tissues, leading to more severe tissue damage. Therefore, there is an urgent demand for wound dressings with antibacterial and anti-inflammatory properties. Considering the concept of moist healing, hydrogels with a three-dimensional (3D) network structure are widely used as wound dressings due to their excellent hydrophilicity, water retention properties, and biocompatibility. Developing antibacterial hydrogels for the treatment of infected wounds has been receiving extensive attention recently. This article categorizes antibacterial hydrogels according to their materials and antibacterial modes, and introduces the recent findings and progress regarding their status. More importantly, with the development of emerging technologies, new therapies are utilized to prepare antibacterial hydrogels such as nanoenzymes, photothermal therapy (PTT), photodynamic therapy (PDT), metal–organic frameworks (MOFs), and other external stimuli-responsive methods. Therefore, this review also examines their progress, challenges, and future trends as wound dressings. In the following studies, there will still be a focus on antibacterial hydrogels that have a high performance, multi-functions, and intelligence, especially biocompatibility, a high and long-lasting antibacterial property, responsiveness, and on-demand therapeutic ability.

## 1. Introduction

Skin, as the largest organ in the human body, plays a significant role in the immune barrier. Generally, wounds can be divided into two types: acute and chronic wounds. For an acute wound, the former type, it forms suddenly and is easy and quick to heal, thus, additional intervention is not essential. However, for a chronic injury (e.g., burn wounds, diabetic ulcers, and pressure ulcers, etc.), it will take several months or even longer to heal and needs appropriate treatment during the entire healing process [1]. Chronic wound sites can not only affect the skin’s structural integrity, but also disrupt the function of barrier protection, thus leading to the easier invasion of pathogenic microorganisms and infection. It is reported that mainly 3–10 dominant microorganisms will accumulate at wound sites and their surrounding environment, including *Staphylococcus aureus* (*S. aureus*), *Streptococcus*, *Pseudomonas*, and anaerobic bacteria, etc. Inappropriate treatment for the bacterial infection at the chronic wound sites will further delay the wound healing process, cause tissue necrosis, and even threaten life. At present, antibiotics are still the most widely used method for infection treatment. However, bacterial resistance has significantly increased with the abuse of antibiotics [2,3,4,5]. Wound dressings are necessarily used to cover the injured sites and protect them from the secondary damage. Therefore, there is an urgent demand for the development of antibacterial and anti-inflammatory wound dressings. 

The ideal wound dressings should have the characteristics of non-toxicity, retaining moisture, and promoting wound healing. Hydrogels with a 3D network structure have a series of excellent properties, such as an excellent hydrophilicity, good water retention, and biocompatibility properties, gaining them much attention for wound healing applications [6,7]. Conventional hydrogels that work as drug carriers containing antibiotics can still not solve the resistant bacteria. To address these problems, developing multi-functional antibacterial hydrogels for the treatment of infected wounds has been receiving extensive attention recently [8,9]. Various methods for preparing antibacterial hydrogels have been studied, including physical cross-linking (e.g., hydrogen bonding, catechol–quinone equilibrium, ionic action, etc.), chemical cross-linking (e.g., covalent, dynamic covalent cross-linking such as thiol-aldehyde addition reaction, and Schiff base, etc.), and multi cross-linking, among which, multi cross-linked hydrogels have the characteristics of elasticity, reversibility, an enhanced strength, and stability by combining several cross-linking approaches. According to the hydrogel materials, antibacterial substances, and antibacterial mechanisms, the available antibacterial hydrogels can be divided into several categories: (1) inherent antibacterial hydrogels [10,11,12], (2) hydrogels functionalized with antibacterial agents [13,14,15], and (3) hydrogels based on new antibacterial therapies [16,17,18]. Briefly, inherent antibacterial hydrogels are those with a natural antibacterial effect without the need for additional antibiotics and antibacterial agents. For the second type, the antibacterial effect of functionalized hydrogels can be endowed through loading antibacterial agents (such as metal ions, nanoparticles, and antimicrobial peptides, etc.) which react and damage the negative charged bacterial surface, finally leading to bacterial death. Compared to the previous traditional types, smart hydrogels based on new antibacterial technologies and therapies have emerged (such as photothermal therapy, photodynamic therapy, and other external stimuli-responsive methods), with a better ability to accelerate wound healing and less adverse effects. 

With the rapid development of polymer materials science and regenerative medicine, more antibacterial substances and antibacterial therapies that promote the optimization of the design and preparation of antibacterial hydrogels have been studied. Compared with the previous studies on antibacterial hydrogels [19,20,21], this review gives an easier and clearer classification of antibacterial hydrogels for this certain application. Benefiting from this, the review provides a more detailed description on their findings, advantages, and problems. In addition, this review selects the suitable studies on these topics in recent five years, details a summary of their achievements and mechanisms, and also looks forward to the future development directions of antibacterial hydrogels in the wound healing field.

## 2. Inherent Antibacterial Hydrogel

Inherent antibacterial hydrogels are a family of hydrogels prepared from materials with intrinsic antibacterial properties. Benefiting from this, these hydrogels can realize antibacterial properties without introducing any additional antibacterial agents or antibiotics. The mechanism of their antibacterial properties mainly depends on the interaction between the cationic groups on the polymer chains and the negative charges on the surface of the bacteria. Generally, inherent antibacterial hydrogels can be divided into natural materials and synthetic materials. 

### 2.1. Natural-Polymer-Based Hydrogels

**Figure 1:**
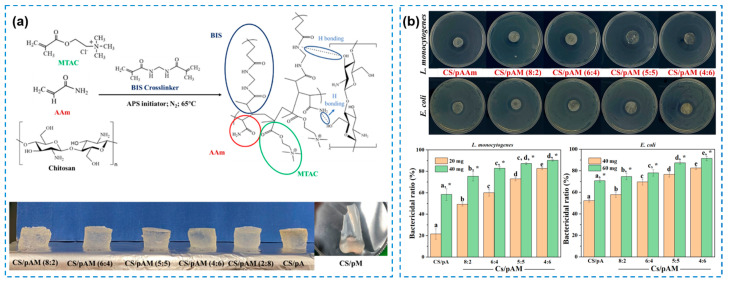
(**a**) Synthesis and preparation of CS-based hydrogels and their optical images. A series of CS/poly(AAm-MTAC) (denoted as CS/pAM) hydrogels with different ratios were prepared through chemical and physical crosslinking. (**b**) Qualitative and quantitative antibacterial characterization of different hydrogels against *L. monocytogenes* and *E. coli* (Different letters a–e indicate significant differences among groups (*p* < 0.05); asterisk (*) presents significant difference within groups (*p* < 0.05)) [11].

Natural polymers are considered to be one of the most suitable candidates in biomedical fields due to their integrated merits of biocompatibility and biodegradability. Natural antibacterial polymers mainly include chitosan, alginate, and their derivatives [22,23], among which, chitosan (CS) and its derivatives are the most widely used natural polymers for preparing inherent antibacterial hydrogels. CS is a co-polymer of D-glucosamine and N-acetyl-D-glucosamine extracted from crustacean animals [24]. The CS-based hydrogel can realize the function of killing bacteria with its positively charged characteristics. Zhu Honglin et al. [11] developed a series of CS-based double-network hydrogels with improved mechanical and antibacterial properties (Figure 1a). The hydrogel was simply constructed by CS and polyacrylamide (pAAm) with the addition of [2-(methacryloyloxy)ethyl]trimethylammonium chloride (MTAC) as the antibacterial agent and *N*, *N*′-methylene bisacrylamide (BIS) as a crosslinker. Their study proved that the composite hydrogels possessed obviously enhanced antibacterial ratios against *Listeria monocytogenes* (*L. monocytogenes*) and *E. coli*, which were nearly 72.85% and 76.60%, respectively (Figure 1b). Actually, the antibacterial effect in this research was mainly attributed to the combination of CS with MTAC. Regarding the antibacterial mechanism of cationic polymers, the difference in the antibacterial efficiency between different bacteria might be attributed to the disparities in their respective cell wall structures of Gram-positive and Gram-negative bacteria. Deng Pengpeng et al. [25] prepared an injectable CS-based hydrogel with self-healing and wound-healing capabilities through an amidation reaction. In brief, the chitosan derivative was created by reacting the amine groups with carboxyl adenine groups. The CS-based hydrogel was then prepared by a heating and cooling process. The obtained CS-based hydrogels showed a high efficiency in terms of antibacterial properties, which was nearly 95.3% for *E. coli*, 97.4% for *S. aureus*, and 100% for *Candida albicans* (*C. albicans*), respectively. However, with an increase in the degree of substitution for the chitosan derivative, the antibacterial effect of the hydrogels gradually decreased, because more amino groups were replaced by adenine groups. Therefore, a suitable degree of substitution is important for the modification of CS to achieve an acceptable antibacterial effect. Their study also confirmed that the hydrogels had no cytotoxicity by showing high cell viability (>100%), strong cell proliferation activity, good hemocompatibility (hemolysis ratio less than 2%), and great clinical application potential as a wound dressing. In addition, as CS has a poor solubility in neutral water, which greatly limits the formation of hydrogels, chemical modification is still necessary to expand its application scope. Besides CS, other natural polymers are also utilized to construct hydrogels as wound dressings. Yu Xiaoshuang et al. [26] used oxidized quaternized guar gum (OQGG) as an antibacterial component by introducing quaternary ammonium groups. The hydrogel could be formed via dynamic covalent cross-linking using carboxymethyl chitosan (CMCS) and OQGG. The antibacterial activity of OQGG was obviously strengthened compared with CMCS due to the enhanced electrostatic interaction between the positively charged OQGG and the negatively charged bacterial cell surface. Furthermore, the in vitro cytotoxicity (75% of cell viability), hemolysis rate (less than 1%), and in vivo bacterial infection wound healing performance indicated that the natural-polymer-based hydrogels had great potential in wound healing. 

Although natural polymeric hydrogels have numerous outstanding gel properties which make them suitable for wound healing applications, the commonly weak mechanical properties of hydrogels in aqueous solutions limit their practical applications. In addition, although natural polymeric antibacterial hydrogels can disrupt bacterial structures by interacting with proteins and phospholipids on their surface via electrostatic action, these hydrogels also have some limitations such as lacking selectivity and long-term efficacy. Therefore, further studies on natural polymeric antibacterial hydrogels are still needed, especially on the aspects of improving their specificity and stability against bacteria. 

### 2.2. Synthetic Polymeric or Hybrid Hydrogels

**Figure 2:**
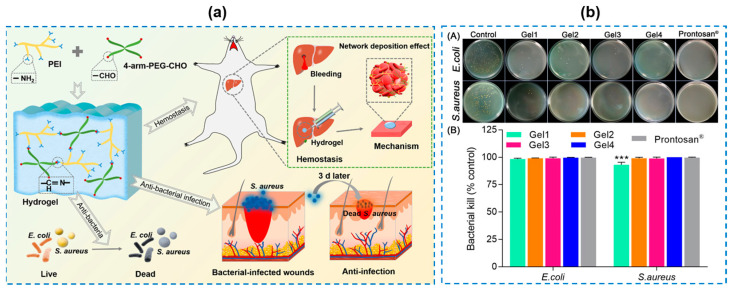
(**a**) Schematic illustration of four-arm-PEG-CHO/PEI hydrogels with hemostatic and antimicrobial capacities. (**b**) Antibacterial activity evaluation of the prepared hydrogels. A: bacterial colonies; B: inhibition ratios against bacteria after incubation for 20 h (Gel1–Gel4 indicated hydrogels with different PEI contents; Gel1 had the lowest PEI contents and Gel4 had the highest PEI contents) [33]. *** *p* < 0.001.

Synthesized materials with inherently antibacterial properties are generally cationic and hydrophilic macromolecular polymers which can form hydrogels by physical or chemical cross-linking [27,28,29]. Inherently antibacterial synthesized polymers mainly include chemically modified polyethyleneimine (PEI), polyacrylic acid (PAA), polyacrylamide (PAM), poly(vinyl alcohol) (PVA), etc. [30,31,32] Hao Yuanping et al. [33] constructed an injectable and self-healing modified PEI with aldehyde groups (-CHO) (named as four-arm-PEG-CHO)/PEI hydrogel applied as tissue adhesives (Figure 2a). In this study, more than 99% of *E. coli* and *S. aureus* bacteria could be eliminated with the help of the hydrogel (Figure 2b). As its antibacterial mechanism, PEI has antimicrobial capacities owing to many amine groups and its inherent polycationic nature. In detail, PEI with cationic protonated amines and many other synthetic cationic polymers can capture and kill bacteria with a negatively charged cell membrane. Instead, with an increase in PEI contents, the cytocompatibility of the composite hydrogel decreased (less than 20% of cell viability). Their study indicated that a suitable PEI content is essential to cytocompatibility, hemocompatibility, and applications for wound care. Generally, more and more synthetic cationic polymeric materials with a good stability and antibacterial long-term effects have been designed recently. However, compared to natural polymeric materials, their cytotoxicity and hemocompatibility should be further considered. For this reason, some researchers prepared composite hydrogels combined with synthetic polymers and natural materials to promote their biocompatibility [34,35,36,37,38,39] (Table 1). Tannic acid (TA) is an amphiphilic tannin existing in a wide range of natural sources. The otriphenol and catechol moieties in TA chains give them excellent antioxidant and inhibitory abilities against both Gram-positive and Gram-negative bacteria. Benefited by the chemical structure containing rich hydroxyl and carboxyl groups, there is a diversity of ways for TA to interact with various synthetic compounds to construct hydrogels via multi cross-linking. Sahiner et al. [40] prepared a linear PEI-based hydrogel by physically and chemically cross-linking with TA. In their study, TA with an antibacterial property was employed as an active biomedical functional agent. The minimum bactericidal concentration (MBC) against both *E. coli* and *Bacillus subtilis* bacteria was determined to be as low as 5 mg/mL. Yu Rui et al. [41] presented a supramolecular gelatin (GT) hydrogel based on GT-graft-aniline tetramer (GT-AT) and quaternized chitosan (QCS). The hydrogel was formed by multi-crosslinking, including host–guest interaction and a dynamic Schiff base. In this hydrogel system, the antibacterial property came from several components. First, QCS was known, as its intrinsic antibacterial properties are due to its abundant quaternary ammonium groups; secondly, GT-AT, as a kind of cationic polymer, kills bacteria for their interaction with anionic bacterial membranes. As a result, the hydrogels exhibited an excellent antibacterial performance with an antibacterial efficiency as high as 91% against *E. coli* and 93% against Methicillin-resistant *Staphylococcus aureus* (*MRSA*), respectively. Additionally, by combining multiple components, the hydrogel exhibited integrated the merits of flexibility, tissue adhesion, self-healing, biocompatibility, antioxidant, and accelerated wound healing. These findings highlight its immense potential in infected wound treatment.

## 3. Antibacterial Hydrogel with Functional Agents

To reduce the risk of wound infection, incorporating functional agents such as metal ions (e.g., zinc and silver), antimicrobial peptides (AMPs), and other bioactive agents into hydrogels is being studied in current research and has proven to be highly efficient in enhancing their antibacterial properties. This part will classify antibacterial hydrogels with functional agents into (1) metal-ions/metal-oxide-loaded hydrogels, (2) antibacterial hydrogels with bioactive agents, and (3) composite hydrogels, reviewing their different types, preparations, and properties.

### 3.1. Metal-Ion/Metal-Oxide-Nanoparticles-Loaded Hydrogels

**Figure 3:**
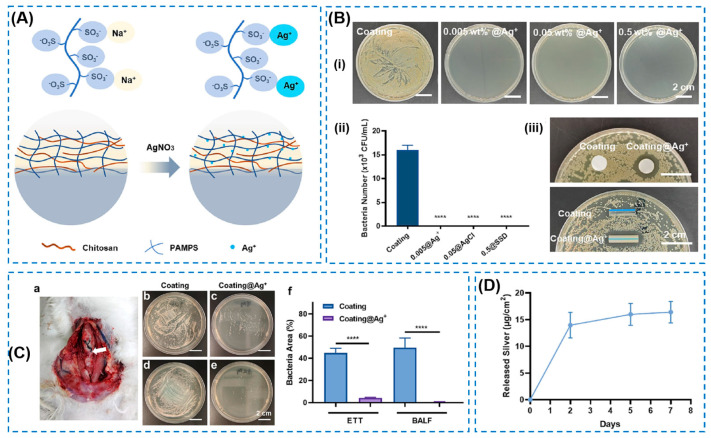
(**A**) Schematic diagram of the silver loading in the hydrogel coating (PAMPS: poly(2-acrylamide-2-methylpropanesulfonic acid)). (**B**) Qualitative and quantitative characterization of bactericidal ability of silver-loaded coatings with various loading amounts of 0.005, 0.05, and 0.5 wt% AgNO_3_. (i: the contact protocol entails co-incubating samples and bacterial suspension; ii: comparison of non-silver-loaded coating and Coating@Ag^+^; iii: the distinct inhibition zone surrounding silver-loaded samples. **** *p* < 0.0001 is considered as statistically significant). (**C**) In vivo anti-infection capability of the coating using a rabbit tracheal intubation model (ETT: endotracheal tube; BALF: bronchoalveolar lavage fluid. a: rabbit tracheal intubation model; bacterial count in ETT eluate cultures of b: the control group and c: the Coating@Ag^+^ group; BALFs cultures of bacterial colonies for d: the control group and e: the Coating@Ag^+^ group; f: the Coating@Ag^+^ group. **** *p* < 0.0001 is considered as statistically significant.). (**D**) Silver release of the silver-loaded coating [48].

Different types of inorganic metal ions (e.g., silver, copper, zinc, and gold ions), metal oxides (e.g., copper oxide and zinc oxide), and their nanoparticles have been investigated as broad-spectrum antibacterial agents to be loaded into hydrogels [15,42,43,44,45]. Some studies on the preparation of metal-ion/oxide-loaded hydrogels are summarized in Table 2 [46,47,48,49,50,51,52,53]. It can be seen that whether in the form of ions or nanoparticles, a high antibacterial efficiency can be achieved. Among different metal materials, silver ions have always been regarded as the most representative antibacterial agent, and have been successfully commercialized in clinical practice [54]. It is known that silver ions can bind to the negatively charged thiol groups on bacterial membrane proteins, leading to protein denaturation and ultimately bacterial apoptosis [55]. Hu Chanchan et al. [56] prepared a silver-loaded coated double-network hydrogel and investigated its anti-infection capabilities at a Ag^+^ concentration of 0.05 wt% (Figure 3A). Their investigations, as shown in Figure 3B,C, demonstrated that this hydrogel was promising in treating healthcare-associated infections. However, the burst release of Ag^+^, especially the in first three days (Figure 3D), may have resulted in their biocompatibility, which was not investigated in this work. Although Ag ions exhibit efficient and broad-spectrum antibacterial activity, their potential cytotoxicity in the body caused by their uncontrolled and direct release profile cannot be ignored. Furthermore, most of the commonly used metal ions have a higher risk of biological toxicity and adverse effects with increases in the concentrations of metal ions. Reducing the amounts of metal ions without decreasing their antibacterial performance poses a major challenge for the fabrication of metal-ion-based antibacterial hydrogels.

Metal nanoparticle (silver, copper, magnesium, and zinc oxides, etc.)-loaded hydrogels have been focused in [15,57]. For example, studies have shown that magnesium oxide nanoparticles (MgO NPs) have the integrated characteristics of inherent stability, biocompatibility, and bioactivity, thus being applied in medicinal and environmental sciences [57]. MgO NPs also display a robust efficacy in killing bacteria, and their antibacterial mechanisms can be attributed to the generation of reactive oxygen species (ROS), including hydrogen peroxide, hydroxyl radicals, and peroxide. Fahaduddin et al. [58] prepared a hybrid hydrogel embedded with green synthesized MgO NPs at a concentration of 10 wt%, and their results confirmed that it was an effective dressing material after antimicrobial efficacy, biodegradability, and anti-inflammatory characterizations. Compared with other types of metal nanoparticles, zinc oxide nanoparticles (ZnO NPs), as a biosafe material, have been proven to be better than other metal oxides due to their better biocompatibility and antibacterial activities over a wide spectrum of bacterial species [49,59,60,61]. Rastegari et al. [57] prepared and characterized a chitosan-based hydrogel loaded with ZnO NPs for controlling the release of vancomycin and enhancing its antibacterial effect. With the assistance of ZnO NPs, the release rate of vancomycin decreased generally. However, the burst release phenomenon was still not solved, as 30% of the drugs could be released within 2 h, which would influence their biocompatibility. The results showed that the obtained hydrogels had a high efficiency against both *S. aureus* and *Pseudomonas aeruginosa* (*P. aeruginosa*). ZnO NPs in this work played an important role in killing bacteria. First, ZnO NPs can act as antibacterial agents; in addition, ZnO NPs are bactericidal by generating ROSs similar to MgO NPs, which can harm the bacterial cell membrane, leading to the release of intracellular contents and ultimately resulting in bacterial death. As a result, the hydrogel had significant and efficient antibacterial activity against *S. aureus* and *Pseudomonas aeruginosa*. 

**Table 2:** Antibacterial hydrogels based on metal ions and metal oxides.

Types of Loading	Types of Metals	Metal Concentration	Antibacterial Ability	Antibacterial Mechanism	Ref.
Metal ions	Silver	50 mM	Notable antibacterial activity against *S. aureus* and *Streptococcus mutants* (*S. mutans*)	Ag^+^ interacts with sulfur-containing proteins in the bacterial cell membrane	[54]
	Copper	1 mg/mL	More than 70% against *E. coli* and *MRSA*	Cu^2+^ generates hydroxyl radicals to attack the bacterial membrane	[56]
	Zinc	4.3 mg/mL	*E. coli*: 99.67%; *S. aureus*: 96.33%	ROS production, lipopolysaccharide membrane rapture, DNA replication inhibition, and lowering the bacteria’s enzymatic metabolism	[57]
Metal nanoparticles	Silver	Ag content: 41.5 wt%	Qualitative analysis: obvious	Released Ag ions can interface with the enzymes and sulphydryl groups of proteins, and inhibit DNA synthesis of the bacteria	[59]
		600 ppm	*Pseudomonas aeruginosa*: 4.20 ± 0.33 log reduction; *MRSA*: 4.56 ± 0.26 log reduction		[55]
	Copper	/	*S. aureus*: 350 μg/mL (MIC) and 1400 μg/mL (MBC); *E. coli*: 500 μg/mL (MIC) and 2000 μg/mL (MBC)	Cu ions damage bacteria cell wall; the reactive hydroxyl radicals prevent the bacterial reproduction and damage of DNA, lipids, and proteins; electrostatic interactions between positively charged Cu ions and negatively charged bacteria	[60]
	Mixture	0.3 wt% (g/mL)	*E. coli*: 98.49%; *S. aureus*: 99.64%	By penetrating the bacterial wall and forming pores on the membrane surface, resulting in cell membrane destruction and leakage of DNA and RNA with cytoplasmic fluid	[58]
		30 wt% of zinc oxide and 5 wt% of hollow silver nanoparticles	Zone of inhabitation of more than 12 mm against *S. aureus* and 2 mm against *Pseudomonas aeruginosa*	Combined mechanism	[61]

### 3.2. Antibacterial Hydrogel with Bioactive Agents

**Figure 4:**
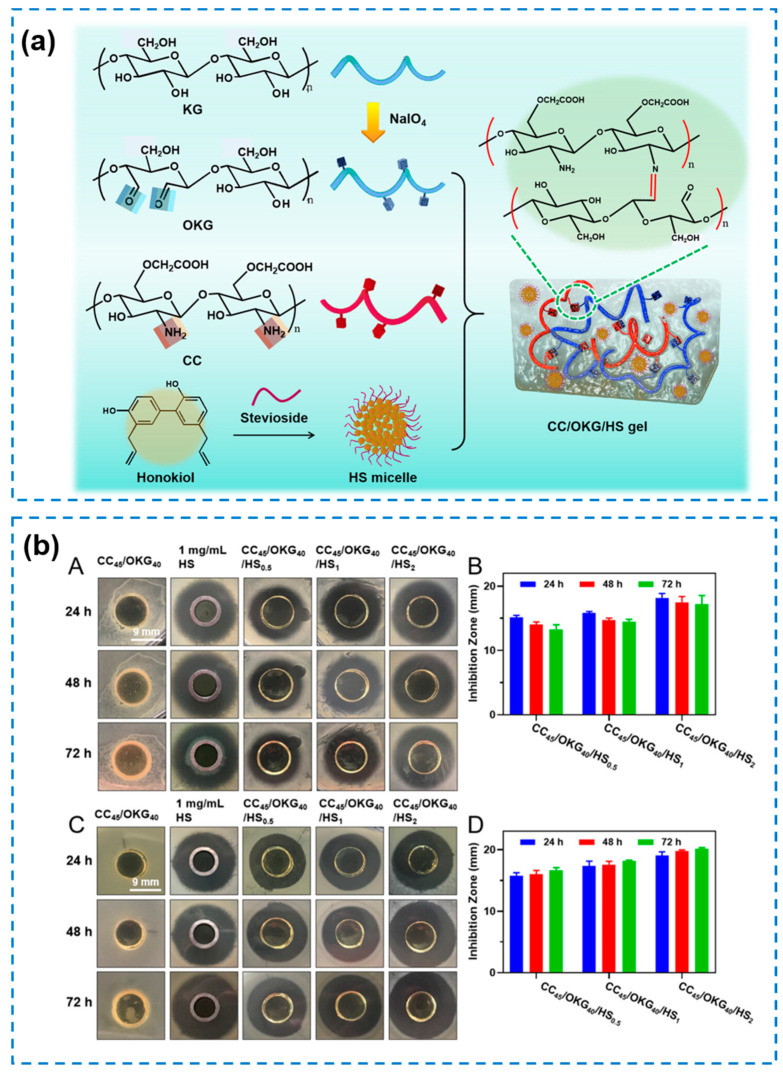
(**a**) Schematic diagram of the preparation of the CC/OKG/HS hydrogels. (**b**) Antibacterial efficiency of the hydrogels (x, y and z in CC_x_/OKG_y_/HS_z_ showed the concentration of CC (x = 45 mg/mL), OKG (y = 100, 80, 60, and 40 mg/mL) and HS (z = 0.5, 1, and 2 mg/mL)) against *S. aureus* (A,B) and *E. coli* (C,D) [14].

Bioactive microspheres/micelles as carriers have been proven effective in antibacterial and wound infection treatment because of their large surface area for a high encapsulation efficiency for antibacterial agents and a high resistance in bacterial cells. Zhang Dongying et al. [62] prepared an oyster peptide (OP)-microspheres-loaded catechol functionalized chitosan (CS-C) and β-glycerol phosphate (β-GP) hybrid hydrogel (named as CS-C/OP/β-GP) with a good biocompatibility and accelerated wound-healing properties. The OPs, composed of active substances such as antibacterial peptides and antioxidant peptides, were proven to have various outstanding biological activities, including antibacterial, antioxidant, and anti-aging, etc. The study proved their biological safety in vivo and in vitro as wound dressings; CS-C/OP/β-GP hydrogels had good procoagulant activity, a high cell viability (more than 100% of cell viability after 24 and 48 h), and a good blood compatibility (less than 3% even under a concentration of 1000 μg/mL). However, assessments of their mechanical and antibacterial properties and potential clinical applications were not performed in their work. In another instance, honokiol is known for its antibacterial, antioxidative, and anti-inflammatory properties, but limited is by its poor solubility and stability in clinical applications. To address this issue, Xu Shuo et al. [14] fabricated a stevioside-stabilized honokiol (HS)-micelles-encapsulated composite hydrogel (labeled as CC/OKG/HS) by chemical cross-linking between carboxymethyl chitosan (CC) and oxidized konjac glucomannan (OKG) (Figure 4a). Their research showed that the CC/OKG/HS hydrogels displayed a HS-amount-dependent antibacterial efficiency attributed to the presence of hoopolyl alcohol against *S. aureus* and *E. coli*, which would be higher when increasing the HS loading amount (Figure 4b). However, a higher HS loading amount would have a negative impact on the biocompatibility. The hydrogels with a lower HS loading amount (0.5 and 1 mg/mL) exhibited an excellent biocompatibility (more than 96% after 72 h). Therefore, it is crucial to select an appropriate concentration of HS micelles. The remarkable antibacterial activity, high biocompatibility, and hemocompatibility exhibited by the hydrogels hold great promise for effectively accelerating wound healing by modulating inflammation.

**Figure 5:**
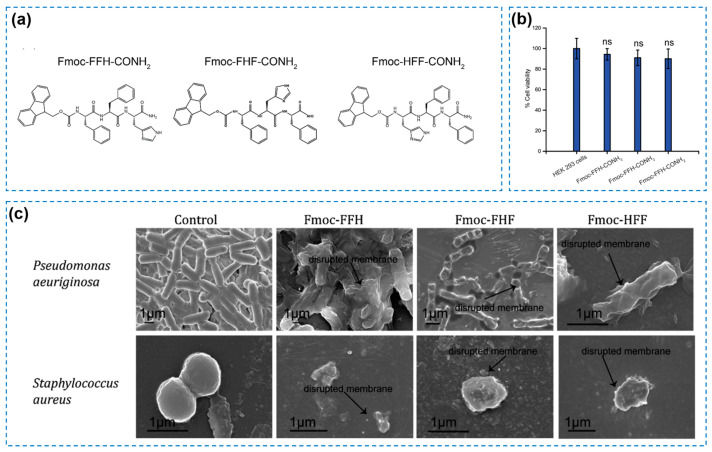
(**a**) Chemical structure of tripeptide: Fmoc-FFH-CONH_2_, Fmoc-FHF-CONH_2_, and Fmoc-HFF-CONH_2_. (**b**) Cell viability of hydrogels (ns means non-significant difference when compared with control). (**c**) The morphologies of disrupted bacterial membrane after hydrogel treatment [69].

**Figure 6:**
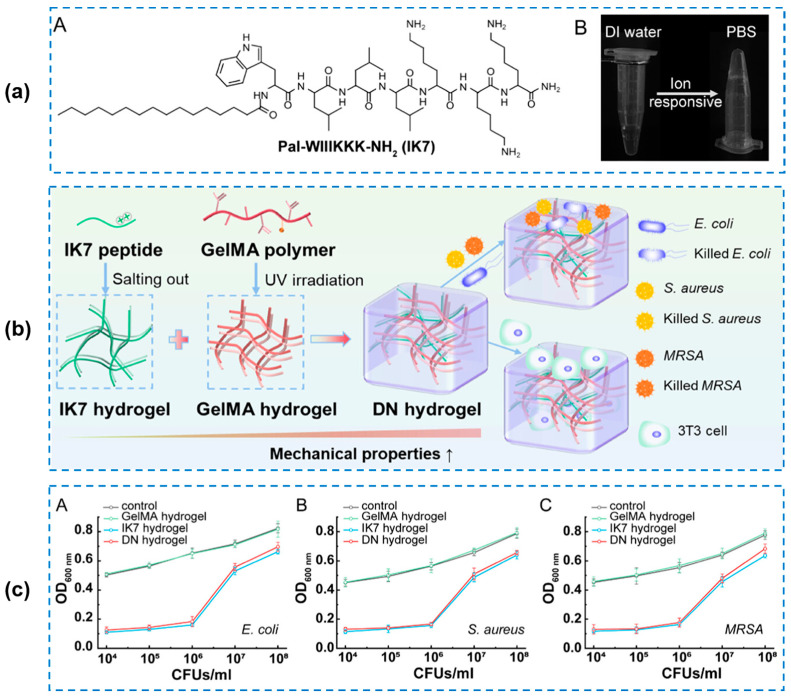
(**a**) Chemical structure of IK7 (A) and inverted vials indicating hydrogel formation (B). (**b**) Formation and properties of IK7, GelMA, and hybrid IK7-GelMA (DN) hydrogels. (**c**) Antibacterial abilities of three hydrogels against *E. coli* (A), *S. aureus* (B), and *MRSA* (C) [74].

**Figure 7:**
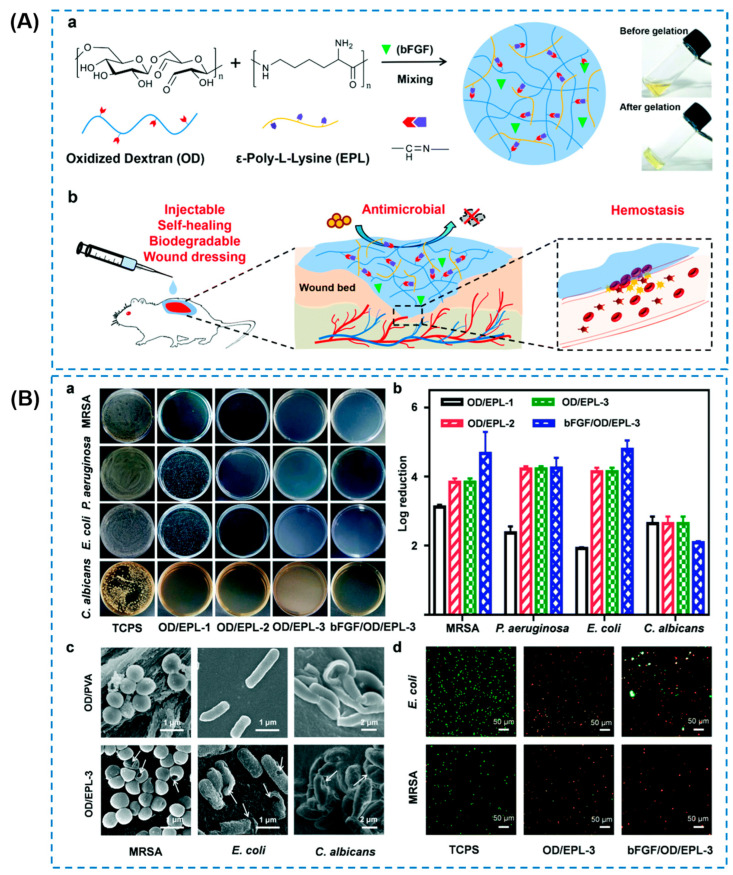
(**A**) Schematic conception for the OD/EPL hydrogels (bFGF: base fibroblast growth factor; a: Schematic illustration of the designed injectable OD/EPL hydrogels; b: The OD/EPL hydrogels possess multiple functions including hemostasis, anti-infection and wound healing). (**B**) Antibacterial activities of OD/EPL and bFGF/OD/EPL hydrogels (OD/EPL-1, OD/EPL-2, and OD/EPL-3 were the hydrogels with the concentration of OD (8%, *w/v* in phosphate buffer saline (PBS) and EPL (6%, 8%, and 10% *w/v* in PBS), respectively) against various bacteria and fungus (TCPS: tissue culture polystyrene; a: optical images of surviving bacterial colonies on agar plates after contact with hydrogels; b: Log reduction of four pathogens on OD/EPL hydrogels; c: SEM images of the morphologies of *MRSA*, *E. coli*, and *C. albicans* on hydrogels; d: LIVE/DEAD bacterial viability assay of *MRSA* and *E. coli*) [76].

Peptide-based hydrogels as alternatives to conventional antibacterial materials have been formulated and proven to be effective in wound treatment [12,63,64,65,66,67]. The primary antibacterial mechanism of peptide-based hydrogels has been explained as the disruption of the bacterial cell membrane structure and the prevention of bacterial regrowth [68]. As a feasible option, peptides are convenient for the construction of supramolecular assembled hydrogels. Prakash et al. [69] designed several tripeptide hydrogels, including Fmoc-FFH-CONH_2_, Fmoc-FHF-CONH_2_, and Fmoc-HFF-CONH_2_ (Figure 5a). The excellent cell viability of cells in Figure 5b indicated their biosafety as wound dressings. The self-assembled hydrogels by the tripeptides showed an inherent antibacterial ability against both Gram-positive and Gram-negative bacteria (Figure 5c). The mechanism of action of these hydrogels was explained as a combination of their β-sheet structure and hydrophobicity. Briefly, the hydrogel would bind to the bacterial membrane and arrange itself to optimize the hydrophilic–hydrophobic interactions, inducing bacteria membrane depolarization. However, this action was less effective for Gram-negative bacteria compared to the Gram-positive one. Except the self-assembled hydrogels, AMPs with positive charges have also been explored to replace traditional antibiotics [70,71]. On one hand, AMPs can inhibit bacterial growth themselves; on the other hand, positively charged AMPs can be easily attracted to negatively charged bacterial cell membranes, disrupt the transmembrane plausible, and destroy the cell membrane, finally killing the bacteria [72]. In addition, most AMPs have been approved for clinical application [73]. Taking advantages of AMPs, Tan Tingyuan et al. [74] designed a peptide C_16_-WIIIKKK (named as IK7, W: tryptophan; I: isoleucine; K: lysine) and then prepared an AMP-based hydrogel (IK7-GelMA) consisting of a photoresponsive gelatin methacryloyl (GelMA) polymer and IK7 by salting out and ultraviolet (UV) irradiation (Figure 6a,b). The obtained hydrogel exhibited a good swelling ratio (nearly 350%) and cytocompatibility (more than 80% of cell viability). The sustained release of the IK7 peptide from the IK7-GelMA hydrogel imbued it with substantial antibacterial ability by cell membrane destabilization, which showed a great potential for next-generation antibacterial hydrogels (Figure 6c). However, their mechanical and practical wound-healing applications were not investigated in this work. For another instance, Gao Fengyuan et al. [75] developed an antimicrobial hydrogel (named as RWPIL-ODEX, R: arginine; W: tryptophan; P: Proline; I: isoleucine; L: leucine; and ODEX: oxidized dextran) based on antimicrobial peptides RWPIL and ODEX, achieving a good cell and blood biocompatibility, and the optimal therapeutic effect against drug-resistant bacteria when using the best biocompatible doses. The RWPIL-ODEX-hydrogel-treated *S. aureus*-infected wound showed an accelerated healing ratio of more than 95% after 15 days. Although AMPs cannot lead to drug resistance, their applications are largely limited by their long process and high costs in production [76]. For this reason, Gao Lingling et al. [76] chose an economical ε-poly-L-lysine (EPL) and prepared a dynamic cross-linked hydrogel (named as OD/EPL) with oxidized dextran (OD) (Figure 7A). The OD/EPL hydrogels showed antibacterial efficiencies of >99.9%, >99.99%, and >99.99% against *MRSA*, *P. aeruginosa*, and *E. coli*, respectively (Figure 7B). The results in this study indicated the immense potential of the hydrogel as a multifunctional wound dressing. 

### 3.3. Composite Hydrogels with Enhanced Antibacterial Property

**Figure 8:**
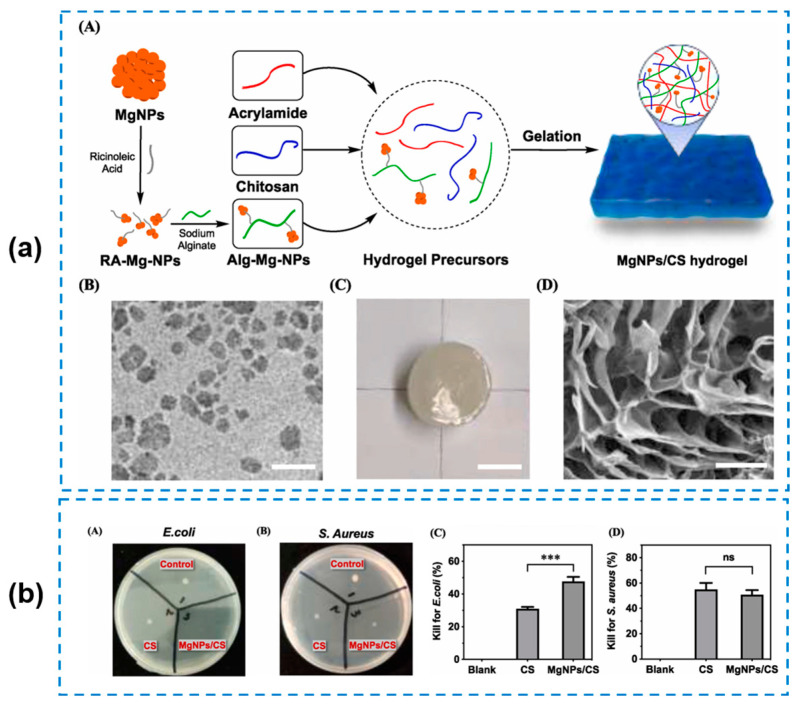
(**a**) A: Synthesis of MgNPs/CS hydrogel, B: morphology of RA-Mg-NPs (RA: ricinoleic acid), C: appearance of MgNPs/CS hydrogel, D: morphology of MgNPs/CS hydrogel. (**b**) Qualitative and quantitative antibacterial activity of MgNPs/CS hydrogel. A: survival *E. coli* clones on agar plate after contacting with hydrogels; B: survival *S. aureus* clones on agar plate after contacting with hydrogels; C: surface antibacterial activity of hydrogels for *E. coli*; D: surface antibacterial activity of hydrogels for *S. aureus*. (one-way ANOVA Tukey test, *** *p* < 0.001; ns: no significant difference) [84].

**Figure 9:**
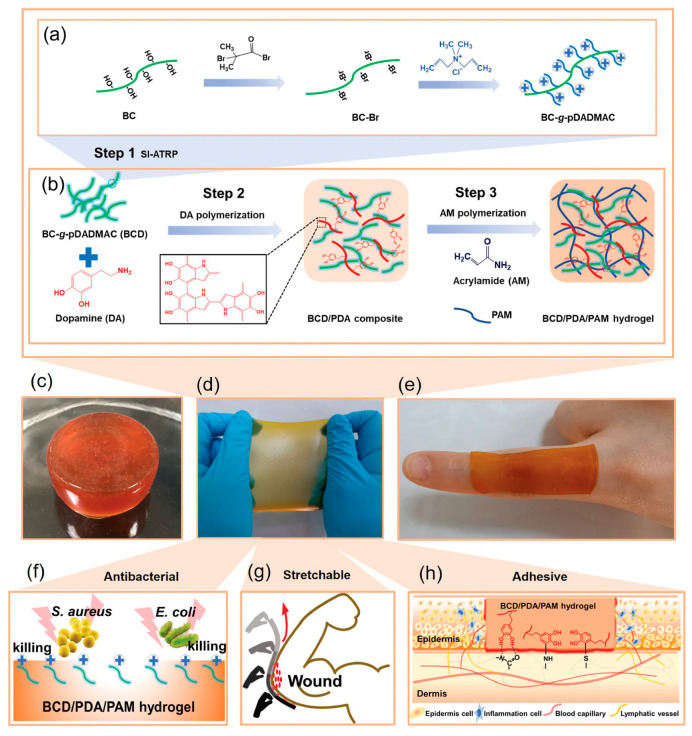
(**a**) Synthesis of BCD. (**b**) Formation of BCD/PDA/PAM hydrogel. (**c**–**e**) Optical images of BCD/PDA/PAM hydrogel. (**f**,**g,h**) Schematic illustration of the antibacterial, stretchable, and adhesive hydrogel [85].

Generally, it is difficult to kill bacteria for severe infections by relying on a single antibacterial effect from inherent antibacterial hydrogel materials. Therefore, more and more composite hydrogel wound dressings have been designed and developed to achieve a synergistic antibacterial activity. Among various studies, introducing drugs as crosslinking agents and chemical modification are the most widely used strategies for preparing hydrogels with synergistic antibacterial activities [77,78]. For instance, Hong Yuanxiu et al. [79] combined AMPs and antibiotics to prepare a polymersome hydrogel composite with long-acting intrinsic antibacterial capabilities. The antibiotic penicillin was encapsulated into the polymersomes, which was then grafted into the hydrogel networks. However, the balance between the cytotoxicity and antibacterial capabilities of the polymersomes was still a noteworthy issue that depended on the drug amount and its release profile. To reduce the risk of released metal ions, ion-crosslinked (such as Ag^+^, Cu^2+^, and Zn^2+^) antibacterial hydrogels have been also prepared in clinical applications for wound healing applications [15,80,81,82]. Meng Weilin et al. [83] designed a mangiferin (MF) self-assembled nanoparticles (MF NPs)-loaded Ag ion-crosslinked hydrogel by utilizing thiourea groups with an excellent ability for metal ion chelation. This method not only provided an easy formation for the hydrogel at a low Ag^+^ concentration, but also achieved a long-term antibacterial property due to its stable release rate of Ag^+^. The obtained hydrogels showed an antibacterial efficiency of more than 99% for both *S. aureus* and *E.coil*, and raising the Ag^+^ concentration could result in a gradient improvement of the antibacterial efficiencies of the hydrogels. Furthermore, with the presence of MF NPs and the reversible property of thiourea-cations chelation, the obtained hydrogels exhibited various advantages such as antioxidative, anti-inflammatory, injectable, and self-healing properties, which are great prospects in the wound-healing field. Qu Jiahao et al. [84] developed a hybrid nanocomposite hydrogel (MgNPs/CS) composed of Mg(OH)_2_ nanoparticles (MgNPs), CS, sodium alginate, and PAM, where MgNPs and CS provided dual-antibacterial activity in this composite system (Figure 8a). As a result, the MgNPs/CS hydrogel showed an antibacterial ratio of nearly 50% both against *S. aureus* and *E. coli*, and compared to the pure CS hydrogel, the integration with MgNPs was proven to be effective in improving the antibacterial activity, especially for *E. coli* bacteria (Figure 8b). In addition, the composite hydrogel exhibited excellent mechanical properties by being soft but tough, able to be stretched to more than 90 times of its original length, and showing a high biocompatibility with a high cell viability (more than 100% at a hydrogel concentration of 1000 μg/mL), which was thought to be an ideal candidate for antibacterial wound dressings. Li Qian et al. [15] developed an antibacterial chitosan/sodium alginate (HCS/SA) hydrogel film embedded with silver nanoparticles (AgNPs) through in situ green reduction with TA with the synergistic antibacterial effect of TA, chitosan, and AgNPs. However, although the introduction of metal ions is effective in endowing the antibacterial property, the toxicity of the released metal ions cannot be ignored. Yang Zifeng et al. [85] constructed a hybrid polydopamine/polyacrylamide (PDA/PAM) hydrogel and incorporated poly(diallyl dimethyl ammonium chloride) (pDADMAC) brushes grafted from bacterial cellulose (BC) nanofibers (BC-g-pDADMAC, BCD). Through the modification of the hydrogels, better antibacterial properties with broad-spectrum and low toxicity can be achieved (Figure 9). In addition, it was proven that the positively charged pDADMAC brushes were helpful for the growth and proliferation of the negatively charged epidermal cells.

## 4. New Therapies for Preparing Antibacterial Hydrogels

### 4.1. Nanoenzyme-Based Composite Antibacterial Hydrogels

Recently, enzymes and enzyme-like materials have shown considerable promise in biomedical field. Enzymes- or their analogues-based wound dressings have been developed with the capacity to catalyze endogenous H_2_O_2_ at the wound site and generate oxygen for accelerating wound healing. Benefited by these characteristics, enzyme-based hydrogels are of great interest for wounds treatment, especially diabetic wounds. It is known that diabetic wounds are difficult to heal owing to their complicated physiological environment, accompanied by various factors like tissue hypoxia, hyperglycemia, and oxidative stress. Moreover, diabetic wounds are more likely to be invaded by pathogenic bacteria. 

**Figure 10:**
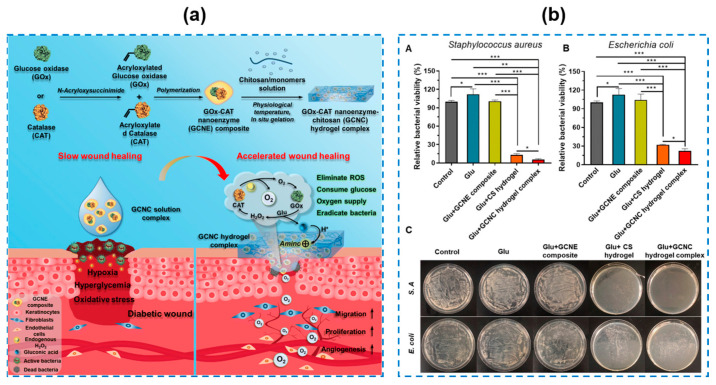
(**a**) The design of GOx–CAT nanoenzyme (GCNE) composite and GOx–CAT nanoenzyme–chitosan (GCNC) hydrogel complex. (**b**) Quantitative evaluation of the antibacterial activity of the hydrogel under different conditions. A: *Staphylococcus aureus* and B: *Escherichia coli* (*** *p* < 0.001, ** *p* < 0.01, * *p* < 0.05); C: representative images of visual bacteria clones (Glu: glucose) [94].

**Figure 11:**
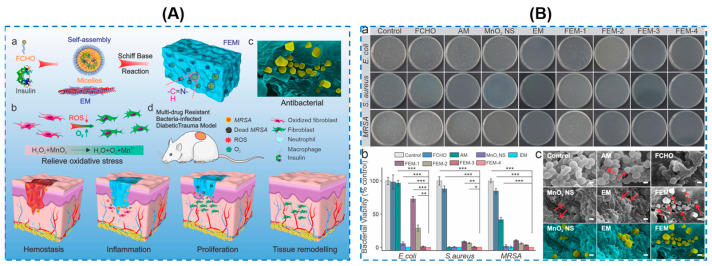
(**A**) Schematic diagram of the preparation and application of FEMI hydrogel (a: The FEMI hydrogel was fabricated by the reversible Schiff-based reaction between EM and insulin-loaded FCHO micelles; b: The FEMI hydrogel could protect fibroblasts from oxidative stress by decomposing the extensive ROS (H_2_O_2_) into O_2_; c: An efficient antibacterial performance was achieved synergistically through the positive-charged EPL and the sharp nanoknife-like MnO_2_ nanosheets; d: The FEMI hydrogel accelerated hemostasis and eradicated MDR infection consumed the extensive deleterious ROS and ameliorated the perpetual inflammatory microenvironment, thus contributing to the stimulated wound healing in vivo). (**B**) Antibacterial performance (a–c) of hydrogel (AM: antibiotic ampicillin; MnO_2_ NS: MnO_2_ nanosheets; FEM-x means different EM contents: 0.05%, FEM-1; 0.10%, FEM-2; 0.15%, FEM-3; 0.20%, and FEM-4; *** *p* < 0.001, ** *p* < 0.01, * *p* < 0.05) [95].

Among enzyme-based therapies, nanozymes, as a kind of nanotechnology therapy, display a broad-spectrum antibacterial ability by catalyzing hydrogen peroxide into highly toxic hydroxyl radicals, which will lead to cell membrane disruption and bacterial death as a result. Futhermore, nanoenzymes can be responsive to changes in the microenvironment at different periods of wound healing, exhibiting variable enzyme-like catalytic activities [86,87,88]. Different types of nanoenzymes, including ferric oxide (Fe_3_O_4_), ceria (CeO_2_), and manganese dioxide (MnO_2_), are utilized, as listed in Table 3 [89,90,91,92]. He Shan et al. [93] reported degradable and biomimetic Ceria (name as CeO_2_–Y@ZIF-8) encapsulated GelMA hydrogels with several positives like antibacterial properties, anti-inflammatory abilities, and wound adaptability. The results showed that over 99.99% for both *S. aureus* and *E. coli* could be killed by the obtained hydrogels after 12 h and an *S. aureus* biofilm formed in vitro could be removed by co-culturing with the hydrogels for 48 h. This study provided a strategy for preparing multi-functional hydrogels for treating diabetic wounds. Li Zhiguo et al. [94] designed an injectable hydrogel based on glucose oxidase (GOx) and catalase (CAT) nanoenzyme-chitosan (GCNC) and applied it for diabetic wound healing (Figure 10a). The prepared hydrogel showed good antibacterial activity against *S. aureus* and *E. coli*; the bacteria viabilities of the hydrogel-treated *S. aureus* and *E. coli* were 5.25% and 22.18%, respectively (Figure 10b). Studies have shown that GOx can catalyze glucose in diabetic wounds to generate H_2_O_2_, which could be subsequently catalyzed by CAT to produce O_2_. After that, the resultant O_2_ could further promote the catalytic activity of GOx, which could activate the amino groups of CS, leading to a better antibacterial capacity. Moreover, a GCNC hydrogel complex had an excellent biocompatibility, even with an increase in the concentrations to 50 μg/mL. As a result, the hydrogel in this research displayed multi-functions and capacities including antibacterial, hemostasis, and the management of blood glucose for diabetic wounds. However, studies suggest it is still difficult to create and maintain a sufficient oxygen concentration at diabetic wounds, limited by inadequate endogenous H_2_O_2_ and the restricted enzymatic activity of individual nanozymes. Wang Shenqiang et al. [95] developed a composite hydrogel (named as FEM and FEMI; FEM means no insulin) utilizing EPL-coated manganese dioxide (MnO_2_) nanosheets (EM) and insulin-encapsulated aldehyde Pluronic F127 (FCHO) micelles (Figure 11A). With an increase in the content of EM, a significant antibacterial enhancement could be obtained (Figure 11B) without sacrificing the cell and blood biocompatibility. An in vivo wound healing experiment showed that the hydrogel had a significantly accelerated wound closure rate by fast hemostasis, eradicating MDR bacteria, relieving oxidative stress, modulating blood glucose levels, and promoting angiogenesis. In their study, MnO_2_ nanosheets used as a nanoenzyme could catalyze the decomposition of the most abundant endogenous H_2_O_2_ into O_2_. The composite hydrogel performed an extraordinary antimicrobial capacity against *E. coli*, *S. aureus*, and *MRSA* via a synergistic combination of positive-charged EPL and “nanoknife-like” MnO_2_ nanosheets (Figure 11B), which was of great potentials for accelerating diabetic wound reconstruction. Up to now, nanoenzymes, as alternatives to antibiotics, are intrinsically limited by their nonbiodegradability and restricted cytocompatibility, thus, further studies on expanding their clinical applications are still urgent.

### 4.2. Photothermal and Photodynamic Antibacterial Hydrogels

**Figure 12:**
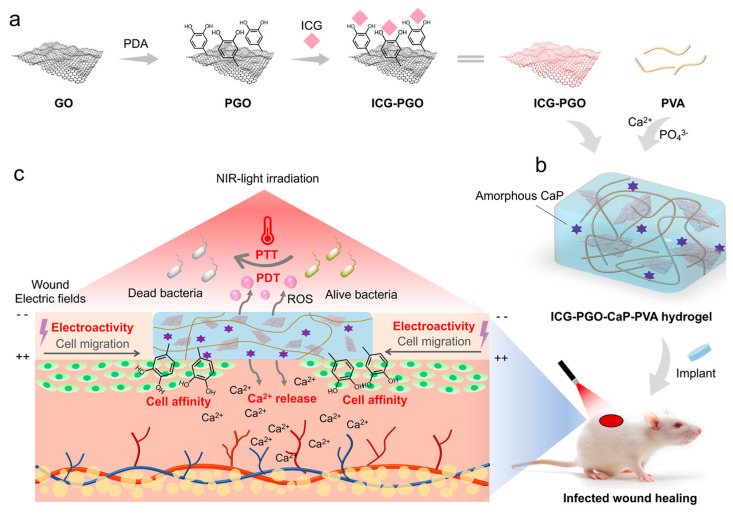
Scheme of the synthesis (**a**,**b**) and mechanism (**c**) of the multifunctional hydrogel for infected wound healing [18].

Infected wound healing is usually accompanied by excessive inflammation, scar formation, and an impaired tissue regeneration ability. Regarding the complex factors, commonly used methods, such as repeated debridement and the long-term use of antibiotics and traditional wound dressings, may impose a huge burden on patients, the consequence of drug-resistant bacteria, as well as an unsatisfactory therapeutic effect. Instead, the most important step for infected wounds is the eradication of bacteria. Most of the used antibacterial agents currently are limited by their low biological activity and potential toxicity, which cannot meet the requirements of tissue regeneration and remodeling during wound healing. In recent years, photothermal therapy (PTT) using nanomaterials as photothermal agents has attracted increasing attention. Combining hydrogels with PTT can not only overcome the shortcomings of traditional methods for wound healing, but also show the advantages of a high efficiency, low irritation, and antibacterial performance. Liu Yingnan et al. [10] constructed a composite hydrogel with an outstanding antibacterial ability by incorporating antimonene nanosheets (AM NSs) with extraordinary photothermal properties into the CS-based hydrogel network. The cell viability of the mouse embryonic fibroblast cells (NIH-3T3 cells) was above 89% after 24 h at the concentration of AM NSs of 0.8 mg mL^−1^. The bactericidal efficiency of the prepared hydrogel under near-infrared (NIR) treatment could reach 97.1% and 100% for *E. coli* and *S. aureus*, respectively, after 10 min. Comparatively, the better bactericidal ability against *S. aureus* was suggested to the better capacity of distinguishing surface charge and cell wall structure. However, the local heat generated may not be sufficient to completely clean the bacteria. For this reason, photodynamic therapy (PDT) is considered to be a promising strategy to kill bacteria synergistically under NIR-light irradiation [17,96,97,98]. Photosensitizers are used to generate ROS upon NIR-light irradiation, which can damage the cell membrane. Chen Yu et al. [16] designed a metal–organic framework composite hydrogel which could generate ^1^O_2_ under NIR laser irradiation and produce less tissue damage. The photodynamic antibacterial efficacy was proven to be remarkably enhanced. However, traditional PDT was greatly hampered by the nonspecific accumulation of photosensitizers, and the generated ROS lacked selectivity, which would lead to the damage of normal cells. To address this issue, Ran Pan et al. [3] developed a hybrid hydrogel (named as PLU@PTc) by conjugating ureido-pyrimidinone on *ε*-polylysine (ePL) and introducing tetrakis(4-carboxyphenyl)porphyrin (TCPP)-loaded PDA (PTc) nanoparticles. The hydrogel exhibited a mechanical performance (mechanical strength of 11.2 kPa), adhesion strength (5.47 kPa), self-healing properties, and the capacity to inhibit bacterial growth for a long time. More importantly, the TCPP in this study reduced the toxicities to normal cells, showing an acceptable cell viability of 77.6% for the PLU@PTc/NIR treatment group. In summary, PDT presents a novel strategy for highly efficient antibacterial therapy for wound healing applications. It is found the combination of PTT and PDT could lead to a synergistic effect of antibacterial and wound treatment. Xie Chaoming et al. [18] reported an approach with the preparation of a multi-functional PVA-based hydrogel (Figure 12). In their study, indocyanine green (ICG) was used as a photosensitizer and grafted onto the surface of polydopamine (PDA)-mediated graphene oxide (PGO) to work as a synergistic PTT/PDT system (ICG-PGO) (Figure 12a); after that, ICG-PGO and calcium phosphate (CaP) were both added in the PVA hydrogel to construct a multifunctional wound dressing (named as ICG-PGO-CaP-PVA) with enhanced mechanical properties, biocompatibility, and a high photothermal and photodynamic efficiency (Figure 12b,c). The composite was able to provide an on-demand antibacterial treatment by generating both local heat and ROS upon NIR light irradiation with few side effects. The results showed that the bacterial survival ratio of the ICG-PGO contained hydrogel reached to 5.6% and 11.3% against *S. epidermidis* and *E. coli*, respectively, after 10 min of irradiation, which was about 17 and 9 times, respectively, compared with the PVA hydrogel. In vivo assessment also confirmed long-term antibacterial efficiency under NIR light irradiation. The hybrid hydrogel could locally convert external radiation into thermal energy and simultaneously generate ROS to kill bacteria. More relevant studies are shown in Table 4 [99,100,101,102,103,104,105]. In summary, PTT and PDT, as emergent therapies, achieved a combined antibacterial effect under NIR-light irradiation by clearing ROS in the early stages of inflammation and proliferation, promoting the polarization and angiogenesis of M2 macrophages and inhibiting excessive vascular growth in the late stage of proliferation and remodeling. In addition, the combined application of PTT and PDT can reduce the temperature required and increase the permeability of ROS compared with an individual therapy. The prepared hydrogels based on PTT and PDT therapies showed significant advantages in preventing drug-resistant bacterial infection and achieving rapid scar free healing, showing good potential in the treatment of drug-resistant bacterial infection wound healing. As bacterial wound infections can occur at any stage during the whole healing process, there is a lot of room for improvements in antibacterial hydrogels based on PTT and PDT therapies, especially for on-demand and long-time antibacterial and excellent wound repair properties in the following studies.

### 4.3. Metal–Organic Framework (MOF)-Based Hydrogels

**Figure 13:**
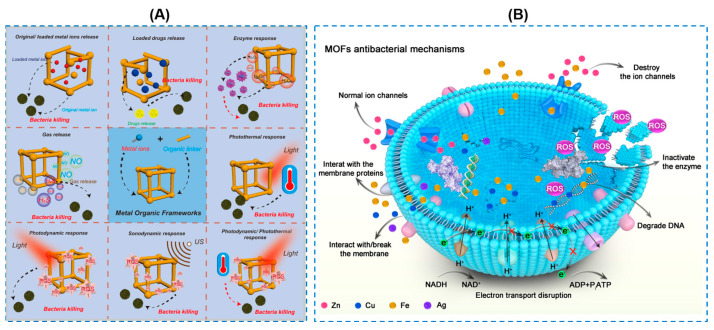
(**A**,**B**) Schematic illustration of antibacterial behaviors of MOFs based on different therapeutic mechanisms [91].

**Figure 14:**
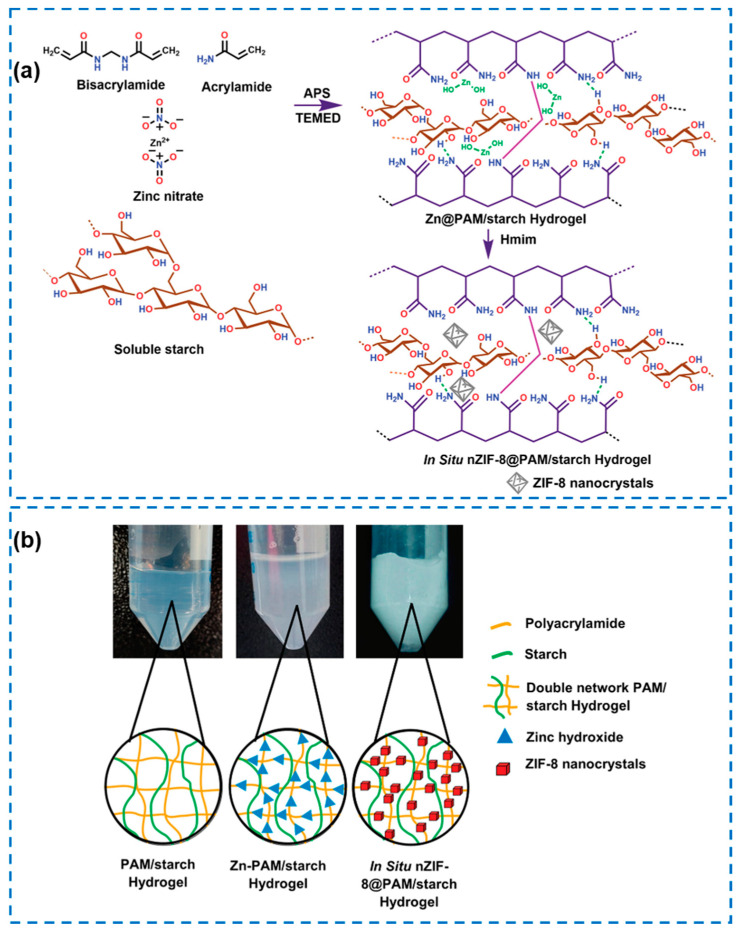
(**a**) Plausible mechanism of the preparation of the in situ nZIF-8@PAM/starch hydrogel (Hmim: 2-methylimidazole). (**b**) Pictorial depiction of the stepwise formation of the in situ nZIF-8@PAM/starch hydrogel [106].

Metal–organic frameworks (MOFs) with a porous structure consist of metal ions with organic ligands [106]. In recent years, MOFs have been considered to be potential candidates as carriers for different applications due to their excellent porosity, surface area, and adjustable size [3,107]. The antibacterial applications of MOFs have been also explored in various studies, and their antibacterial properties come from several components based on different therapeutic mechanisms, as illustrated in Figure 13 [108]: (1) the metal ions released from MOFs; (2) the organic ligands in the MOF structure; and (3) MOFs as platforms for loading functional materials or antimicrobial agents. Among their diverse families, zeolitic imidazolate framework-8 (ZIF-8), with a good chemical and thermal stability, high porosity, and outstanding biocompatibility, are one of the most suitable candidates for biomedical applications. In addition, ZIF-8 exhibits a high photocatalytic and broad-spectrum bactericidal activity. Moreover, the incorporated ZIF-8 is beneficial for improving the mechanical property of the composites due to the existence of multicomponents in the MOF structure [3]. Yao Xiaoxue et al. [109] reported an emulsion templating strategy to obtain a ZIF-8 loaded omniphobic hydrogel. This composite could prevent bacterial invasion and enable the controlled release of the antibacterial Zn ions simultaneously. However, this preparation was reported to be involved in complex synthetic procedures. Reddy et al. [106] proposed a smart method for incorporating nano-ZIF-8 (nZIF-8) into a polyacrylamide/starch hydrogel (PSH) by in situ growth (Figure 14). The hydrogel had a transmittance value of 80%, sufficiently allowing light to pass. The nanoarchitectonics of the nZIF-8-based hydrogel in their study combined the merits of self-adhesion, an improved mechanical strength, and photodynamic therapy, and worked as a sustained-release drug delivery carrier in the PSH hydrogel, showing great potential in the application for topical treatment. Li Na et al. [110] designed and synthesized a type of modified ZIF-8 nanoparticles by loading polyhexamethylenebiguanide (PHMB) into nanoparticles (denoted as P-ZIF), where PHMB could be released in the weak acid environment of an infected wound. P-ZIF was then encapsulated into an injectable hydrogel consisting of sodium alginate (SA) and 3-aminophenylboronic-acid-modified human-like collagen (H-A). As it antibacterial mechanism, the PHMB and Zn ions in P-ZIF could be released for a synergistic antibacterial effect. 

As most MOFs achieve antibacterial capacity by releasing bioactive metal ions or ligands during the decomposition process of metal–ligand bonds, the problem of potential toxicity caused by excess released metal ions will happen inevitably. To solve this issue, increasing the stability of metal ions has become an important point when designing MOF-based hydrogels. Ameer et al. [111] chose copper MOFs, a more robust antibacterial agent than other MOFs, to design a copper MOF nanoparticles-encapsulated hydrogel with a stable and sustained release ratio. The hydrogel system exhibited a lower copper release amount due to the cooperative effect between the copper MOF nanoparticles and poly-(polyethyleneglycol citrate-co-Nisopropylacrylamide) (PPCN)-based matrix. In addition, the hydrogel could accelerate wound healing by promoting collagen deposition, cell migration, and angiogenesis [112,113]. To further investigate the therapeutic effectiveness of the MOFs-contained dressings in detail, different types of MOFs, including Cu-, Co-, and Zn-based materials, were synthesized, respectively, and their covering MOF-loaded hydrogels were prepared by Gwon et al. [112] The capacities of the MOF-embedded hydrogels were tested against *E. coli* and *S. aureus*. The results showed physical properties such as that the surface area and dimension of MOFs with different central metals appeared to be more important than the chemical properties of the ligands in determining the effects on bacteria, and Cu- and Co-embedded hydrogels displayed an excellent antibacterial activity among the three types, especially the Cu-MOF, which could kill 99.9% bacteria with no cytotoxicity. More relevant studies on the MOF-based hydrogels are listed in Table 5 [114,115,116,117]. Up to now, as a high concentration of MOFs will result in cytotoxicity through the excessive release of metal ions that oxidize proteins, DNA, and lipids, controlling the release of metal ions under physiological conditions to improve its biosafety is still required.

### 4.4. Other External Stimuli-Responsive Smart Hydrogels

**Figure 15:**
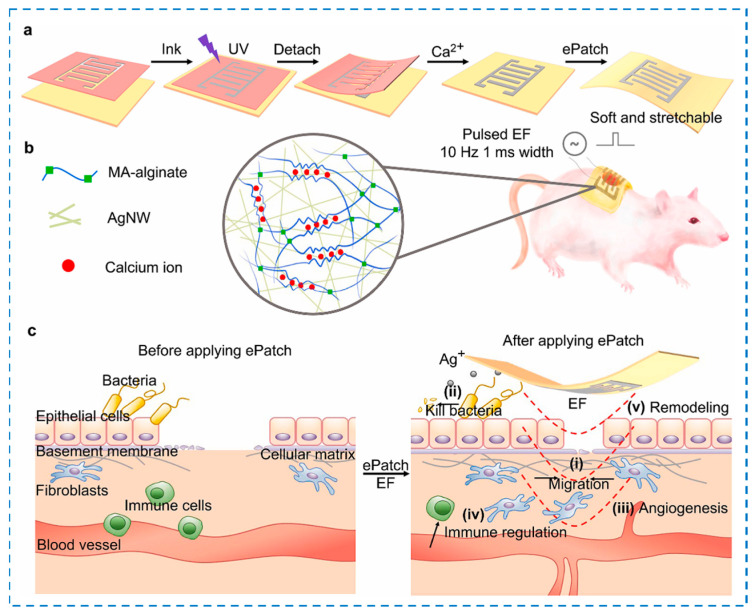
(**a**,**b**) Schematic of the ePatch fabrication. (**c**) Illustration of the biological activities of the ePatch during the healing process.

**Figure 16:**
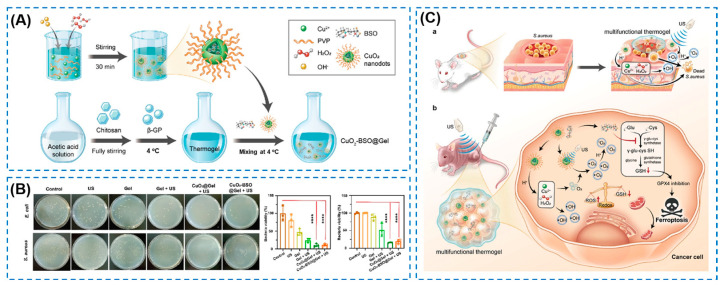
(**A**) Scheme of the preparation of CuO_2_-BSO@Gel. (**B**) In vitro antibacterial performance (**** *p* < 0.0001). (**C**) Mechanism diagram of CuO_2_–BSO@Gel for bacteria-infected wound healing.

Other methods have been developed to prepare external stimuli-responsive smart hydrogels for treating wounds such as pH-, ultrasound-, electrical-, and biomolecule-responsive, and sonodynamic therapies [118,119]. Compared with traditional hydrogels, smart hydrogels as wound dressings can interact with wounds and even be responsive to changes in the injured environment. Considering that the wound sites displayed a distinct pH condition (pH 4.5–6.5), Wu Ye et al. [120] utilized phenylboronic acid (PBA)-grafted oxidized dextran (denoted as POD) and caffeic acid (CA)-grafted *ε*-polylysine (denoted as CE) to construct a hybrid hydrogel (named as POD/CE) by a dynamic Schiff base and boronic ester bonds. The hydrogel showed an excellent degradability, a high water absorption rate, injectability, and self-healing properties as wound dressings. Additionally, the hydrogel exhibited an excellent antibacterial ratio against *S. aureus* (91.1%) and *P. aeruginosa* (97.3%) due to the release of the CE polymers. Furthermore, the hydrogel with a pH/ROS dual responsive property was more beneficial for healing chronically infected diabetic wounds by showing more effective release behavior in an acidic and ROS-abundant environment. Electrical field (EF) stimulation is used for accelerating wound healing regarding its limiting adverse effects. On the one hand, the delivery of EF at the wound site can activate ion channels, which is beneficial for guiding the migration and proliferation of epithelial cells and fibroblasts; on the other hand, EF will induce angiogenesis and immune modulation in wound healing. Wang Canran et al. [121] prepared a flexible electric patch (named as ePatch) in which silver nanowire (AgNW) and methacrylated alginate (MAA) were formed as the hydrogel layer to deliver EF stimulations to the wound (Figure 15a,b). With the assistance of EF generators in this work, the ePatch combined several advantages as wound dressings, including suppressing bacteria growth and preventing bacterial infection, accelerating fibroblast migration and proliferation, as well as promoting blood vessel formation, re-epithelization, and tissue remodeling (Figure 15c). The constructed EF-assisted system was indicated to be greatly promising for facilitating wound healing in the clinic. In addition, sonodynamic therapy (SDT) is also considered to be a noninvasive wound healing treatment by activating sonosensitizers under ultrasound irradiation to produce ROSs. Zheng Yaling et al. [122] developed an injectable chitosan hydrogel (named as CuO_2_–BSO@Gel) by integrating CuO_2_ nanodots and L-Buthionine-(S, R)-sulfoximine (BSO) (Figure 16A). As ultrasound has a strong tissue penetration ability, the sonosensitizer in the CuO_2_–BSO@Gel can be effectively stimulated to produce sufficient ROSs. Furthermore, copper irons also had a superior antibacterial activity. As a result, most of the bacteria treated with CuO_2_–BSO@Gel could be killed (Figure 16B). The multifunctional CuO_2_–BSO@Gel with synergistic chemodynamic therapy (CDT)-SDT effects were promising for eradicating melanoma and bacteria-infected wound healing (Figure 16C). In summary, although smart hydrogels with external stimuli-responsive properties show a great potential in wound healing applications, they are always accompanied a complex fabrication process and multiple components which will produce potential biological toxicity and need high costs. Therefore, the development and commercialization of smart hydrogels with biosafety and a high performance will be the main future direction. 

## 5. Conclusions, Challenges, and Prospects

Hydrogels are of great interest in constructing multifunctional wound dressings because of their various properties, such as their 3D network structure, high permeability to water and oxygen, and biocompatibility. In this review, we focused on the recent research and performed a review on the progress of antibacterial hydrogels for the wound healing application, especially for infected wounds. Generally, we classified antibacterial hydrogels into inherent antibacterial hydrogels (including natural and synthetic polymeric hydrogels), antibacterial hydrogels with antibacterial agents (such as metal ions, bioactive agents, and AMPs), and other hydrogels using modern antibacterial therapies (such as PTT and PDT). The inherent antibacterial hydrogels have the advantages of wide sources, a low cost, and simple preparation, but their antibacterial effect may be limited. With antibacterial agents, antibacterial hydrogels show better and dual antibacterial activity, providing new therapeutic strategies for alternative antibiotic therapies. However, their potential cytotoxicity cannot be ignored. New antibacterial therapies are now considered to be one of the most promising ways to resist wound infection, with the advantages of being non-invasive, environment responsive, and having a high efficiency. 

Despite these advances, antibacterial hydrogels as wound dressings still face some major challenges. (1) As a protective material for wound sites, hydrogels should have enough mechanical properties including tensile, compressive strength, and self-healing properties. Although some of the studies in this review noticed the mechanical properties of antibacterial hydrogels, the mechanical properties of hydrogels still need to be strengthened. More design methods on the hydrogel structures, such as double cross-linking structure and fiber reinforcement, can be used to improve the mechanical properties of hydrogels. (2) The antibacterial properties, including the efficiency and durability, are still limited by the single hydrogel material or single mechanism. Multicomponent and multi mechanisms can be utilized in constructing the antibacterial hydrogel system to achieve a longer and sustained antibacterial property. (3) Multi-functions are necessary to improve the potential clinical application of hydrogels, e.g., biocompatibility, antioxidant, anti-inflammatory, and cell proliferation promotion, to enhance their therapeutic potential. (4) The commercialization of antibacterial hydrogel dressings to promote wound healing is another huge obstacle. Therefore, it is still challenging to develop an antibacterial multi-functional hydrogel with commercial potential.

Concerning the challenges, the future prospects of antibacterial hydrogel dressings are proposed as follows. (1) There is still a lot room for inherent antibacterial materials, which are considered to be one of the most direct methods in the antibacterial field. Developing new materials with a lasting and broad-spectrum antibacterial effects will still be the key point in the following studies. (2) Smart antibacterial hydrogels such as micro-environment-responsive, on-demand release, and real-time diagnosis are the trend to improve the antibacterial and therapeutic efficacy. (3) More functions and basic properties should be developed and enhanced for an antibacterial hydrogel wound dressings, especially for mechanical properties, anti-bleeding, self-healing, and therapeutic effects, to cure skin wounds during entire process.

## Figures and Tables

**Table 1:** Examples of hybrid polymeric hydrogels and their properties.

Components	Antibacterial Mechanism	Antibacterial Ability	Other Performance	Ref.
PVA/PAA/TA	TA interrupts the biological activity of bacteria	Obvious inhibition zones for *S. aureus* (11.5 mm) and *E. coli* (9.83 mm)	Self-healable, elastic, highly toughness, tissue-adhesive, hemostatic	[37]
polyethylene oxide (PEO)/guar gum (GG)/rosemary (RM)/citric acid (CA)	RM attacks the cell membrane of bacteria, subsequently diminishing bacterial cell growth	Obvious inhibition zones for *S. aureus* (9 mm) and *E. coli* (2 mm)	Moisture adsorption, hydrophily, and cell attachment and proliferation	[38]
PAA/L-lysine derived branched polyetheramides (Lys-BPEA)	Lys-BPEA rupture of the bacteria cell membrane	The inhibition rate against *E. coli* and *S. aureus* exceeded 80%; long-term stable antibacterial activity	Good mechanical strength, self-healing, and no obvious hemolytic behavior	[39]

**Table 3:** Examples for nanoenzymes used for wound care.

Types of Nanoenzymes	Other Components	Antibacterial Property	Other Performance as Wound Dressings	Mechanism of Action	Ref.
Fe_3_O_4_	TA	*No data*	Blood compatibility, antioxidative ability, excellent therapeutic efficacy, promote wound healing	Catalyzing the decomposition of H_2_O_2_ to generate nontoxic products in neutral environment or to generate hydroxyl radical in acidic environment	[89]
CeO_2_ nanorods	Oxide dextran/*ε*-polylysine (EPL)	Broad-spectrum antibacterial activity (almost 100%) against *E. coli*, *S. aureus*, and *MRSA*	Self-healing behavior, good adhesiveness, hemostatic ability and promoted MRSA-infected diabetic wound healing	Affecting bacteria plasmalemma and physiological metabolism; releasing antibacterial EPL	[90]
PDA@MnO_2_	Polydopamine (PDA)/thioctic acid/TA	Notable antibacterial activity against *E. coli* and *S. aureus*	Injectable, self-healing, adhesive, biocompatible, antioxidant, anti-inflammatory, and promoted the chronic diabetic wound healing	Scavenging various types of reactive nitrogen and oxygen species, and generating O_2_ by degrading H_2_O_2_	[91]
MnO_2_ nanosheets	Poly(ethylene glycol) methyl ether methacrylate (PEGMA)/glycidyl methacrylate (GMA)/acrylamide (AAm)	Broad-spectrum antibacterial activity against *MRSA*, *E. coli* and *Pseudomonas aeruginosa* (as high as 99.9%)	ROS-scavenging, O_2_ generation, anti-oxidative, accelerated the infected diabetic skin wound healing	Decreasing the level of ROS, suppressing the inflammation and neutrophil infiltration, and promoting the polarization of macrophages into M2-type	[92]

**Table 4:** Examples for hydrogel wound dressings on the basis of PTT and PDT.

Therapy Type	Functional Agents	Concentration	Antibacterial Ability		Antibacterial Mechanism	Ref.
PTT	Graphene oxide (GO)	3–5 wt%	*E. coli* and *S. aureus*: 99.9%		The use of photothermal reagents to convert light energy into heat energy	[99]
	CuS nanoparticles (CuSNPs)	2 mM	Qualitative analysis: obvious			[100]
	Protocatechualdehyde (PA)	4.5 mg/mL	*E. coli*: 89.2%; *S. aureus*: 87.0% (1 min); *E. coli* and *S. aureus*: 100% (10 min)			[101]
PDT	Ag/Ag@AgCl	/	*E. coli*: 95.95%; *S. aureus*: 98.49%	Photosensitizers (PSs) combined with light and oxygen can generate ROS		[102]
	Black Phosphorus (BP)	/	*E. col*: 98.90%; *S. aureus*: 99.51%			[103]
	Berberine Chloride (BBR)	312.5 μM	*E. coli* and *S. aureus*: 625.0 μM (MIC); *E. col*: 79.1%; *S. aureus*: 100%			[104]
PTT/PDT	iron-doped carbon dots (Fe-CDs)	/	*E. coli* and *S. aureus*: more than 99%	Fe-CDs can act as photothermal therapeutic agents and photodynamic therapeutic agents to generate heat (approximately 50 °C) and rapidly catalyze the decomposition of hydrogen peroxide to produce hydroxyl radicals		[105]

**Table 5:** Studies on different MOF-based hydrogel as wound dressings.

Types of MOF	Metal Ions	Antibacterial Ability	Ref.
Cu-MOFs	Cu^2+^	99.9% against *S. mutans* and MRSA; 78.7% against *C. albicans*	[115]
K-MOF	K^+^	Qualitative analysis: obvious	[116]
Co-MOF	Co^2+^	Higher long-lasting antibacterial effect	[117]
ZIF-8	Zn^2+^	Qualitative analysis: obvious	[118]

## Data Availability

No new data were created.
